# The Use (Not Abuse) of Nitrous Oxide Gas

**Published:** 1872-04

**Authors:** Arthur C. Ford

**Affiliations:** Atlanta, Ga.


### ARTICLE II

### *The Use (not abuse) of Nitrous Oxide.*

BY ARTHUR C. FORD, D.D.S., ATLANTA, GA.

Our personal and esteemed friend, Dr. S. J. Cobb, of Nashville, contributed an article to the February number of your Journal, entitled "A Naughty Case," in which we think he makes some broad and *Naughty* observations with regard to the use of Nitrous Oxide, and quotes our distinguished and honored fellow practitioner, Prof. S. P. Cutler, of New Orleans, as an authority who abets him in his opinion that said Nitrous Oxide is a "great humbug," and ought to be entirely eschewed by our profession. We beg to join issue with the doctor as to the propriety of entertaining this proposition of abolition ; and while we condemn the abuse of this "Gas," and particularly for the wholesale extraction of teeth by men who, we blush to acknowledge, are called dentists, yet we have seen a large number of cases where the administration of an anæsthetic was not only expedient but absolutely necessary, and where the judicious administration of Nitrous Oxide, was, in our opinion, for many reasons much to be preferred to the employment of any of its cotemporaries. All persons, it is well known, are not constituted alike, and though *we* may be able to tolerate the pain of extraction, yet we must not, and indeed need not, expect to find every person endowed with the same fortitude as ourselves, and frequently where we find extraction imperative, we must be prepared by some means to extend to our patients immunity from pain or give them "Dutch courage," and we think it preferable to resort to Gas than to alcoholic stimulants, as frequently was the case in years gone by, and indeed still is in places where Gas is inaccessible. Nitrous Oxide is, we believe, the most convenient anæsthetic for dental purposes, and when carefully generated, and administered with judgment, decidedly the most harmless; we do not say there is *no* danger in its administration, but after five years experience with it we can say, with a clear conscience, that we have never yet "extracted the wrong tooth, crazed our patient, or sent him to his long home," nor do we know of a solitary case wherein one of our patients received the least injury from its use. The only objection we have to Gas is, not its danger, but that it is too much trouble to the operator, that is to a practitioner of dentistry—we do refer to those Colton Institutes where its administration is made a specialty—and for this reason we endeavored at one time to do away with our Gasometer, but our patients were so clamorous for its assistance that we were obliged to resume operations; we would say in passing that it is far from being a paying institution with us, we keep it solely for the accomodation of our patrons. The mere fact that there have been some unfortunate results attending the administration of this anæsthetic, I believe *hardly one in one hundred thousand*, a very small proportion, is no argument against its use; if it is, it is also effective against many other necessaries and conveniences, viz: people ought not travel by rail, stage coach, horseback, steam boat, etc. etc., *ad infinitum;* why? Because so many have lost their lives by so doing.

Many persons have died under the hands of the surgeon, whilst anæsthetics were employed, and sometimes even in minor cases of surgery; are we to insist therefore, that, all men hereafter *must* suffer under the knife, and not be permitted to take advantage of these annihilators of pain, the parties interested being willing to run the risk of a fatal termination, dreading the operation as much as death itself? Why the very idea is absurd, untenable, and would be hooted at by three-fourths of mankind. Anæsthetics have become necessaries to civilized man, and so will continue until some less dangerous and equally efficatious method is discovered. A small body of men cannot successfully combat the popular will in such matters.

To illustrate the necessity of applying to the *use* of Gas occasionally, we will relate a case in practice we had a few days ago.

A lady applied to us to have the roots of the right second inferior molar removed. They were very much ulcerated. and there was a fistulous opening a little forward of the angle of the ramus, that had been discharging for about a year. In addition to her being remarkably nervous, she labored under the additional disadvantage of a deformity or peculiar formation, that precluded the possibility of her opening her mouth sufficiently wide to admit of any instrument, capable of removing the roots; moreover, said roots were so sore she would not permit of their being touched, The crown had been broken off by a country dentist, while endeavoring to extract the tooth, he not having control of his forceps on account of this inability to open the mouth.

We administered the Gas and by the assistance of a wooden wedge of soft pine, forced the mouth open sufficiently, the muscles being somewhat relaxed, to admit of a small pair of forceps, and removed the offending roots. The patient recovered in a few minutes and if Dr. C. could have beheld her joy, relief and thankfulness, we think he would have acknowledged that Nitrous Oxide was not "a great humbug." If Gas had not been used, a resort to some other anæsthetic would have been absolutely needful. The lady's brother-in-law is a physician, and being present, concurred with me that the recourse to some such agent was imperative.

Now the "*Naughty*" part of the doctor's article, we consider consists in the extreme view he takes against anæsthetics in general, and Nitrous Oxide in particular, as applicable to our speciality in surgery. We have ever found that there is a "medium in all things;" everything in this world has its use, it is only the abuse of an article that causes it to be pernicious. The doctor also lays great stress upon "truth." In this we coincide with him and believe that "truth is omnipotent," etc., and would here endeavor to impress upon him the *truth* of the old adage that "extremes are dangerous," and would therefore recommend him to leave the ranks of the extremists, those who advocate too boldly, and also those who abuse too roundly any "person, place or thing," and get into the middle of the stream with the "middle men," and he will be apt to find with them both truth and "Mr. Grant's" electioneering countersign "peace."

